# Integrating AI and Assistive Technologies in Healthcare: Insights from a Narrative Review of Reviews

**DOI:** 10.3390/healthcare13050556

**Published:** 2025-03-04

**Authors:** Daniele Giansanti, Antonia Pirrera

**Affiliations:** Centro TISP, ISS, Via Regina Elena 299, 00161 Rome, Italy; antonia.pirrera@iss.it

**Keywords:** assistive technology, artificial intelligence, AI, machine learning, deep learning

## Abstract

The integration of artificial intelligence (AI) into assistive technologies is an emerging field with transformative potential, aimed at enhancing autonomy and quality of life for individuals with disabilities and aging populations. This overview of reviews, utilizing a standardized checklist and quality control procedures, examines recent advancements and future implications in this domain. The search for articles for the review was finalized by 15 December 2024. Nineteen review studies were selected through a systematic process identifying prevailing themes, opportunities, challenges, and recommendations regarding the integration of AI in assistive technologies. First, AI is increasingly central to improving mobility, healthcare diagnostics, and cognitive support, enabling personalized and adaptive solutions for users. The integration of AI into traditional assistive technologies, such as smart wheelchairs and exoskeletons, enhances their performance, creating more intuitive and responsive devices. Additionally, AI is improving the inclusion of children with autism spectrum disorders, promoting social interaction and cognitive development through innovative devices. The review also identifies significant opportunities and challenges. AI-powered assistive technologies offer enormous potential to increase independence, reduce reliance on external support, and improve communication for individuals with cognitive disorders. However, challenges such as personalization, digital literacy among the elderly, and privacy concerns in healthcare contexts need to be addressed. Notably, AI itself is expanding the concept of assistive technology, shifting from traditional tools to intelligent systems capable of learning and adapting to individual needs. This evolution represents a fundamental change in assistive technology, emphasizing dynamic, adaptive systems over static solutions. Finally, the study emphasizes the growing economic investment in this sector, forecasting significant market growth, with AI-driven assistive devices poised to transform the landscape. Despite challenges such as high development costs and regulatory hurdles, opportunities for innovation and affordability remain. This review underscores the importance of addressing challenges related to standardization, accessibility, and ethical considerations to ensure the successful integration of AI into assistive technologies, fostering greater inclusivity and improved quality of life for users globally.

## 1. Introduction

Assistive technologies (ATs) play a crucial role in supporting individuals with disabilities by enhancing independence and quality of life. According to the National Institute of Child Health and Human Development (NICHD), AT includes a wide range of devices, from basic prosthetics to advanced mobility and communication aids, addressing physical, cognitive, and sensory challenges [1]. The NICHD highlights the need for affordable and effective solutions, particularly in low-resource settings [1].

The European Disability Forum (EDF) emphasizes AT’s role in enabling social, educational, and professional participation, advocating for policies that ensure equitable access regardless of financial or geographical constraints [2]. Similarly, the National Cancer Institute recognizes AT’s significance in healthcare, helping patients, including those with cancer, manage daily activities and improve their quality of life [3].

The World Health Organization (WHO) defines AT as

“*Assistive technology is an umbrella term for assistive products and their related systems and services Assistive products help maintain or improve an individual’s functioning related to cognition, communication, hearing, mobility, self-care and vision, thus enabling their health, well-being, inclusion and participation*” [4]. A broad definition with also the exclusion criteria is defined in a document by ISO 9999:2022 (available online: https://www.iso.org/standard/72464.html, accessed on 7 February 2025).

The growing demand for assistive technologies (ATs), projected to reach over 2.5 billion people by 2050, highlights the urgent need for inclusive policies to overcome economic and infrastructural barriers, particularly in low- and middle-income countries [4]. Over the past decades, AT has evolved significantly, transforming accessibility and inclusion across multiple domains [5]. From the 1980s onward, technological advancements have continuously shaped AT. The introduction of personal computers brought essential tools like screen readers and speech synthesis, enabling digital accessibility for visually impaired users [6,7]. The rise of the Internet in the 1990s further expanded opportunities, facilitating real-time subtitles and web accessibility [8,9]. The early 2000s saw the proliferation of smartphones and apps, integrating features such as screen readers, voice assistants, and real-time translation, which significantly enhanced communication and mobility for individuals with disabilities [10,11,12]. More recently, artificial intelligence (AI) and robotics have propelled AT into new frontiers, with innovations like smart prosthetics, exoskeletons, and brain–computer interfaces providing unprecedented levels of autonomy [11,12]. The impact of AT is evident across diverse applications, addressing the needs of individuals with varying disabilities [8,9,10,11,12]. Visual accessibility technologies, such as screen readers and Braille devices, have revolutionized access to information, while auditory aids, including real-time captions and augmentative communication tools, have enhanced social and professional participation. For individuals with motor disabilities, electric wheelchairs, prosthetics, and adaptive control systems offer greater independence. Cognitive and communication disabilities are supported by educational software, memory aids, and speech synthesis technologies, facilitating interaction and learning. Meanwhile, neuromotor innovations, such as exoskeletons and neural control systems, are redefining rehabilitation and mobility for individuals with severe impairments.

As AT continues to evolve, ensuring equitable access remains a global priority. Policymakers must address affordability and infrastructure gaps to harness the full potential of these technologies, fostering a more inclusive and accessible society.

ATs continue to evolve, driven by innovations in AI, robotics, and the Internet of Things (IoT), creating new opportunities to improve inclusion and autonomy for people with disabilities in every area of daily life. As we move forward, the integration of AI into ATs is significantly enhancing their capabilities.

AI is “*a technical and scientific field devoted to the engineered system that generates outputs such as content, forecasts, recommendations or decisions for a given set of human-defined objectives*” [ISO/IEC 22989:2022] (available online: https://www.iso.org/artificial-intelligence/what-is-ai, accessed on 7 February 2025).

AI is revolutionizing ATs, making devices more adaptive, intelligent, and accessible. AI-powered solutions, such as OrCam’s MyEye and dot Lumen glasses, enhance autonomy by providing real-time assistance for the visually impaired [13]. AI-driven tools like ChatGPT 4 and Verbit improve accessibility for individuals with cognitive and linguistic disabilities, facilitating communication and education [14].

In higher education, AI supports students with attention deficit hyperactivity disorder (ADHD) by enhancing focus, time management, and organization, ensuring greater inclusivity in learning environments [15]. The integration of AI with the Internet of Things (AIoT) is further transforming AT, enabling wearables and smart home systems to provide real-time, personalized support for mobility, health monitoring, and social interaction [16].

AI is not only enhancing traditional AT but is also becoming an assistive technology itself, as it enables smart, anticipatory, and highly personalized solutions [14,15]. By redefining accessibility and inclusion, AI-driven AT is shifting from passive support tools to dynamic, responsive systems that empower individuals with disabilities in all aspects of life [16]. This evolution extends to healthcare, where AI improves diagnostics and intervention strategies, as for example for conditions like autism, fostering more tailored and effective solutions [17,18].

### 1.1. Purpose

The integration of ATs and artificial intelligence AI is becoming increasingly critical in enhancing accessibility and improving the quality of life for people with disabilities. The convergence of these technologies offers transformative potential across healthcare, education, and daily living environments, addressing a diverse range of needs. Understanding how AI can be leveraged to optimize assistive devices and systems is crucial to unlocking these opportunities. A narrative review of review studies (NRR) is valuable because it synthesizes comprehensive analyses from reviews based on multiple studies, rather than focusing on individual studies. Reviews aggregate a wide range of research, highlighting stable themes and trends, and offering a clearer understanding of AI-driven AT advancements. They provide a broader, more balanced perspective and indirectly reveal knowledge gaps, making them ideal for identifying emerging areas, policy implications, and opportunities for further exploration in the field.

The purpose of this study is, therefore, to perform a narrative review of reviews to analyze the current state of integration between ATs and AI, with the specific objectives to:

- *Analyze the Growth of Research in AT and AI*

Examine the overall increase in research output on assistive technologies and AI, focusing on the volume and scope of publications.

- *Identify Established Themes and Categories*

Identify key areas of focus in recent reviews, such as AI applications in assistive devices, the role of machine learning and deep learning, and advancements in diagnostic and therapeutic processes.

- *Examine Opportunities and Challenges*

Explore the potential benefits and challenges of integrating AI into assistive technologies and extract the key direct and indirect recommendations.

### 1.2. Structure and Organization of the Review

The Introduction Section 1.2 is organized to provide a clear framework for the study, starting with an Introduction section that introduces the research topic, establishes its significance, and outlines the primary objectives. This sets the stage for understanding the context and purpose of the investigation.

Next, the methods Section 2 describes the research approach, explaining how data were collected and analyzed to ensure transparency and reproducibility in the study.

The study results Section 3 is organized into three main sections:

- Section 3.1 examines bibliometric trends, focusing on publication volumes over time, with an emphasis on the last 5 and 10 years. This highlights the growing interest and development of AI in assistive technologies.
- Section 3.2 analyzes the selected review studies on AI applications in healthcare-related assistive technologies. It categorizes the emerging themes, providing a deeper understanding of the key areas of innovation and research in the field.
- Section 3.3 identifies emerging opportunities for further research and development based on the study’s findings, presenting key recommendations for advancing AI in assistive technologies.

The discussion Section 4 is structured into four parts:

- Section 4.1 summarizes the key findings and outcomes from the review.
- Section 4.2 delves into trends, opportunities, and challenges in AI-driven assistive technologies, offering actionable recommendations.
- Section 4.3 explores future perspectives, building on the recommendations and reviewing recent studies that continue to shape the field.
- Section 4.4 highlights the limitations of the study, providing a balanced view of the research.

Finally, the conclusions Section 5 wraps up the study by summarizing the key insights and their broader implications for the future of AI in assistive technologies.

### 1.3. Synoptic Visual Overview of Study Structure and Key Findings

**Figure 1:**
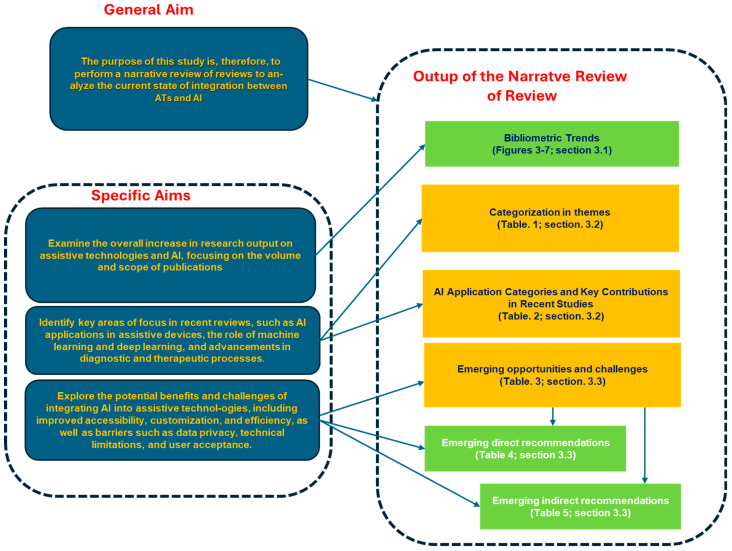
First synoptic diagram visual representation.

**Figure 2:**
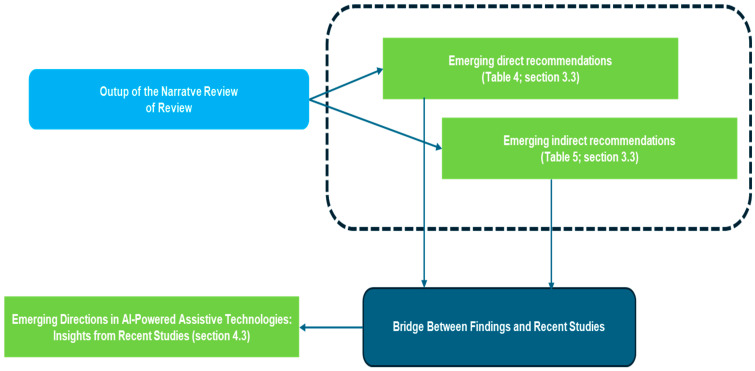
Second synoptic diagram visual representation.

**Figure 3:**
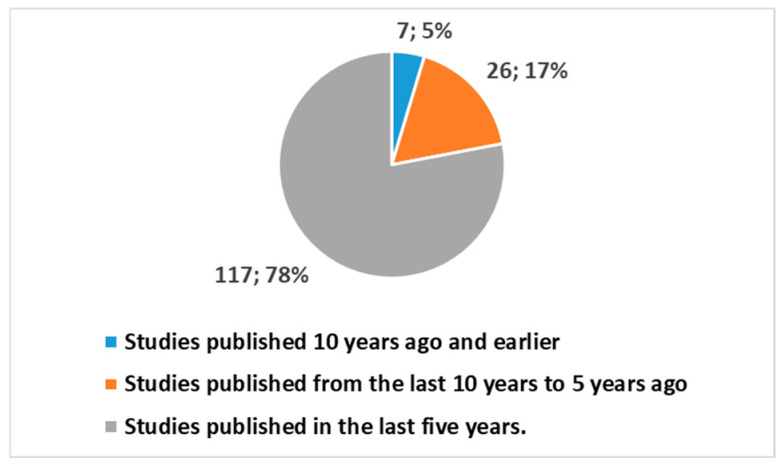
Trends of publications in different periods using the key in position 1 of Box 1.

**Figure 4:**
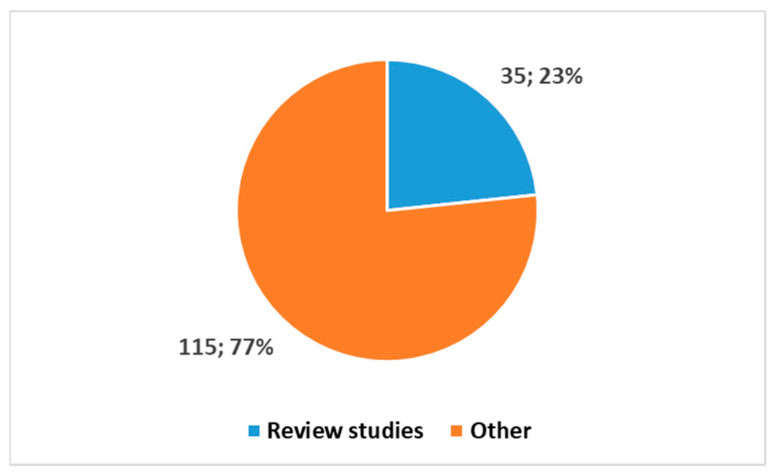
Proportion between review studies and other papers in the search using the key in position 1, Box 1.

**Figure 5:**
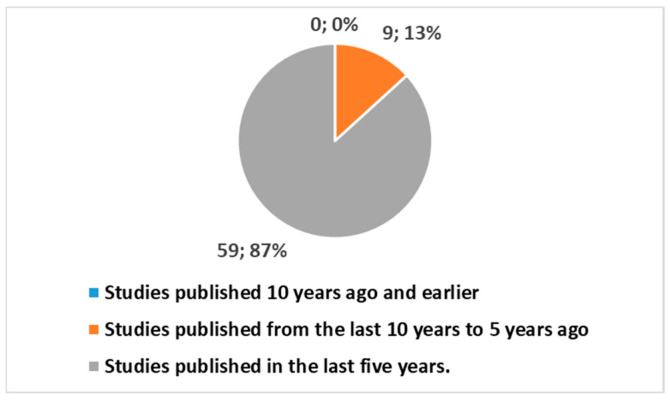
Trends of publications in different periods using the key in position 2 of Box 1.

**Figure 6:**
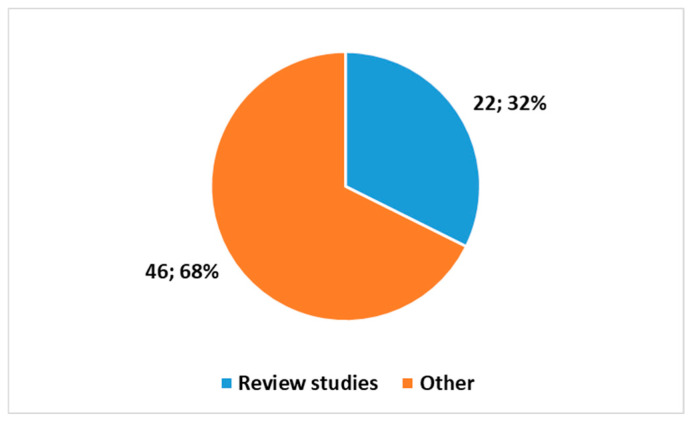
Proportion between review studies and other papers in the search using the key in position 2, Box 1.

**Figure 7:**
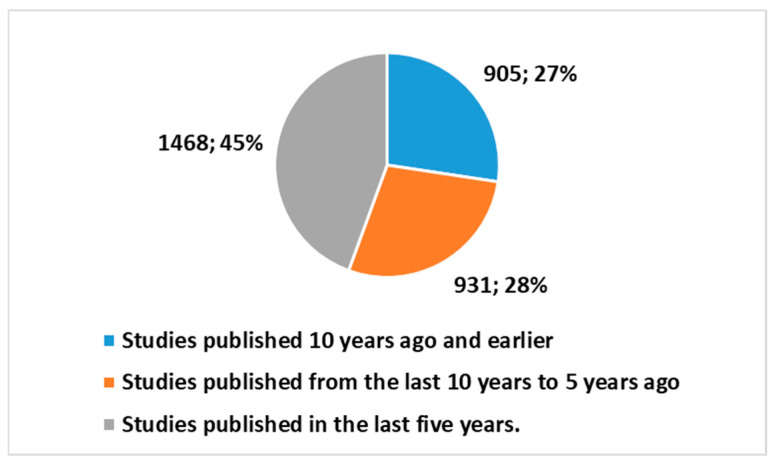
Trends of publications on ATs in different periods.

A synoptic diagram based on two figures (Figure 1 and Figure 2) is reported for the sake of clarity. It helps in understanding the presentation of the results and the interpretation of the discussion as reported in Figure 3, Figure 4, Figure 5, Figure 6 and Figure 7 and Table 1, Table 2, Table 3, Table 4 and Table 5.

The study’s structure is visually represented in Figure 1, which follows a top-down approach to illustrate how the findings are organized. At the top (Block 1), bibliometric trends related to AI applications in assistive technologies are presented, as detailed in Figure 3, Figure 4, Figure 5, Figure 6 and Figure 7 in Section 3.1. Moving down (Block 2), the studies are categorized by thematic areas (refer to Table 1 in Section 3.2), organizing AI applications into key domains. In the lower middle (Block 3), a comparative analysis of these AI applications (Table 2 in Section 3.2) refines their classification, showcasing different use cases. At the bottom (Block 4), emerging opportunities and challenges (Table 3 in Section 3.3) are summarized, noting the benefits of AI, such as improvements in autonomy and diagnostics, as well as challenges like ethical concerns and accessibility barriers.

Figure 2 connects these findings to the discussion. Block 1 (Top Right) presents the direct recommendations (Table 4, Section 3.3), which focus on specific improvements for AI in assistive technologies. Below Block 1, Block 2 explores the indirect recommendations (Table 5, Section 3.3), addressing broader issues such as regulation, collaboration, and standardization. Finally, Block 3 (Bottom Left) highlights cutting-edge research directions (Section 4.3), identifying emerging areas that align with the earlier recommendations. This visual structure creates a clear bridge between the findings and actionable insights, ensuring a smooth progression from immediate recommendations to broader systemic considerations and future research directions.

## 2. Methods

This review of reviews used a standardized checklist designed for the narrative category of reviews (see [19]). The narrative review was performed based on targeted searches using specific composite keys on PubMed and Scopus. The search for articles for the review was finalized by 15 December 2024.

The overview was also conducted using a qualification methodology based on proposed quality parameters described in [20] to decide the inclusion of the study in the overview, focusing on its application in healthcare, the field of interest of the journal, and the following algorithms.

See Algorithm 1 used in the literature overview.
**Algorithm 1.** The proposed algorithm for the overview of reviews.Set the search query to:*“(assistive technology [Title/Abstract]) AND ((artificial intelligence [Title/Abstract]) OR (machine learning [Title/Abstract]) OR (deep learn-ing[Title/Abstract]) OR (neural network [Title/Abstract]) OR (ANN [Title/Abstract]) OR (AI [Title/Abstract]) OR (GANN [Title/Abstract]) OR (CNN [Title/Abstract]))”*Conduct a targeted search on Pubmed and Scopus using the search query from step 1.Select studies published in peer-reviewed journals that focus on the field. Priority is given to the most recent reviews, particularly when they incorporate and critically assess previous studies on the same topic. This approach ensures that the selection reflects the most updated and comprehensive synthesis of the available evidence, reducing redundancy and highlighting the most current advancements in the field. When multiple reviews exist on a given subject, preference is given to those that provide a broader analysis, integrate findings from earlier works, and offer new perspectives or insights, while maintaining scientific rigor and methodological soundness.For each study, evaluate the following parameters:N1: Is the rationale for the study in the introduction clear?N2: Is the design of the work appropriate?N3: Are the methods described clearly?N4: Are the results presented clearly?N5: Are the conclusions based and justified by the results?N6: Did the authors disclose all the conflicts of interest?Assign a graded score to parameters N1–N5, ranging from 1 (minimum) to 5 (maximum).For parameter N6, assign a binary assessment of “Yes” or “No” to indicate if the authors disclosed all the conflicts of interest.Preselect studies that meet the following criteria:Parameter N6 must be “Yes”.Parameters N1–N5 must have a score not lower than 3.Include the preselected studies in the overview.From the process of selection, the following review studies were identified [21,22,23,24,25,26,27,28,29,30,31,32,33,34,35,36,37,38,39].The scoring output is reported in the Supplementary Material.

## 3. Results

The study results have been organized into three main sections to provide a structured and in-depth overview.

*Section 3.1:* Focuses on bibliometric trends in this field by analyzing the progression of publication volumes over time, with a particular emphasis on the last 5 and 10 years. This offers insights into the evolution and growing interest in the topic.

*Section 3.2:* Examines the selected review studies, which focus on the application of artificial intelligence (AI) in assistive technologies for healthcare [21,22,23,24,25,26,27,28,29,30,31,32,33,34,35,36,37,38,39]. This section explores emerging themes and provides a categorization to better understand the key areas of research and innovation.

*Section 3.3:* Based on the findings from the analyzed studies, it identifies emerging opportunities, highlights areas that require further exploration and development to drive significant advancements in the field, and provides key recommendations for future directions.

This systematic approach provides a clear and comprehensive view of the state of the art, current trends, and future prospects in the application of AI to assistive technologies in healthcare.

### 3.1. Trends in the Publications in the Intersection of AI and Assistive Technologies

Including an analysis of bibliometric trends in a NRR is essential for contextualizing the field’s evolution through both a statistical and narrative approach. Statistically, analyzing publication growth over the past 5 and 10 years using trend graphs and reviewing study proportions helps quantify the field’s expansion and maturity. Narratively, interpreting these trends provides insights into research dynamics, emerging areas, and gaps, guiding future investigations and contextualizing the growing interest in the topic.

A bibliometric analysis was conducted using the composite search key outlined in position 1 of Box 1 to explore trends in PubMed studies. This search yielded 150 studies since 2007, of which 35 are reviews, including systematic reviews.

Interestingly, narrowing the timeframe highlights the following trends (Figure 3):

- 143 studies (26 + 117), accounting for 95.3%, were published in the last 10 years;
- 117 studies, or 78%, were published in the last 5 years.

Figure 4 reports the proportion of reviews. Nearly all the review studies were published within the last decade (34 out of 35), with 30 reviews, representing 85.7%, appearing in the last 5 years. This reflects a growing interest in synthesizing and critically evaluating the available literature, a hallmark of a field reaching consolidation and maturity.

A second search was performed using a search key limited only to “artificial intelligence” (e.g., without using *other key such as machine learning, deep learning*) paired with assistive technologies (position 2 of Box 1). This search includes articles that address AI in a general sense, without necessarily specifying the techniques employed (e.g., machine learning, expert systems, or classical algorithms). Rather than encompassing a broad range of technologies and applications, it takes a more focused approach, concentrating on how AI is implemented in AT rather than on how specific algorithms optimize solutions.

This query retrieved 68 studies, all published since 2015, and thus produced in the last 10 years (Figure 5). Of these: 59 studies, or 86.8%, were published in the last 5 years.

Figure 6 reports the proportion of reviews in this second search.

A further search using key 3 from Box 1, focusing only on ATs without AI, yielded 3304 studies, with these studies comprising the following:

- 2399 studies (or 72.6%) published in the last 10 years;
- 1468 studies (or 44.4%) published in the last 5 years.

Figure 7 reports these trends.

When we compare the research trends, we observe a stark contrast between studies that integrate AI with assistive technologies and those that focus solely on assistive technologies (ATs). The search using the broader keys for “artificial intelligence” and “assistive technologies” (positions 1 and 2 in Box 1) identified a growing body of research in this area, particularly in the past decade.

This comparison shows a remarkable difference in the rate of publication. Studies that include AI in assistive technologies have seen a much steeper rise in recent years. Specifically, the proportion of studies published in the last 5 years is much higher for AI-related research using key 1 in Box 1 (86.8% for studies with AI versus 44.4% for studies on ATs alone). Similarly, the growth over the last 10 years is also more pronounced for AT and AI studies (95.3% vs. 72.6%).

This trend is even more striking when considering the research on AI and AT using key 2 in Box 1, which shows 100% of studies were published in the last 10 years and 86.8% in the last 5 years.

Results highlight that AI is rapidly becoming a central component of research in assistive technologies, driving innovation and expanding the scope of possible applications.

Box 1 **Used composite keys.**

*(assistive technology [Title/Abstract]) AND ((artificial intelligence [Title/Abstract]) OR (machine learning [Title/Abstract]) OR (deep learning [Title/Abstract]) OR (neural network [Title/Abstract]) OR (ANN [Title/Abstract]) OR (AI [Title/Abstract]) OR (GANN [Title/Abstract]) OR (CNN [Title/Abstract]))*

*(assistive technology [Title/Abstract]) AND (artificial intelligence [Title/Abstract])*

*(assistive technology [Title/Abstract])*

### 3.2. Themes and Categorization

Table 1 presents emerging themes in AI applications across various domains, including healthcare, education, assistive technology, and cognitive health. The studies highlight AI’s role in enhancing diagnosis, navigation, robotics, and human–machine interactions. Key themes include AI-driven assistive technologies for disabilities, emotion recognition, and brain–computer interfaces, showcasing AI’s impact on improving accessibility and healthcare.

Table 2 categorizes AI applications into key areas such as robotics, medical diagnosis, assistive technologies, cognitive health, and communication aids. Each study demonstrates AI’s contributions, from improving surgical precision and diagnostic accuracy to enhancing mobility, cognitive function, and emotional support, emphasizing AI’s transformative role in multiple fields. The studies included in these tables are also analytically summarized in the Supplementary Material (see Text S1. Analytical summary of the studies).

**Table 1:** Emerging themes.

Reference	Description	Focus	Role of AI	Theme
Kokorelias et al. [21]	This scoping review explores the coadaptation process between older adults and smart technologies (wearables, voice-activated virtual assistants).	Understanding the evolution of interactions between older adults and smart technologies over time, focusing on technology integration and user experience.	Smart technologies like virtual assistants involve AI for interaction and personalization.	Aging, Assistive Technology, Smart Devices
Vistorte et al. [22]	A systematic review of how AI is used to assess emotions in educational environments.	AI-driven emotion recognition in education, exploring its effects on learning and student well-being.	Machine learning, facial recognition, and AI techniques are used to assess and interpret emotional states to improve educational outcomes.	Education, Emotion Recognition, AI Integration
Okolo et al. [23]	Review of assistive technologies for visually impaired individuals, focusing on navigation aids.	Improving mobility and navigation for visually impaired individuals through assistive technology.	AI plays a role in developing assistive systems like vision-based navigation tools and multimodal support.	Disability, Assistive Technology, Navigation
Eldawlatly [24]	Review of the role of generative AI in developing brain–computer interfaces (BCIs).	Overcoming challenges in BCI systems through generative AI, improving data resolution and user performance.	AI is used to enhance the capabilities of BCIs, generating synthetic brain data to improve system performance and expand applications.	Brain–Computer Interfaces, Generative AI, Assistive Technology
Wei et al. [25]	Exploration of AI applications in skin cancer diagnosis and screening.	AI’s role in diagnosing and screening skin cancer, from image processing to molecular analysis.	AI aids in image and molecular data analysis, improving diagnosis and screening accuracy for skin cancer.	Healthcare, Skin Cancer, AI Diagnostics
Xie et al. [26]	Review of robot-assisted laparoscopic surgery in gynecology, focusing on the role of robotics.	Investigating the use of robot-assisted technologies in gynecological surgeries, comparing it with traditional methods.	AI assists in enhancing the precision and efficiency of robotic laparoscopic surgery in gynecology.	Medical Robotics, Surgery, Assistive Technology
Iannone and Giansanti. [27]	Analysis of AI integration in assistive technology for autism care.	Examining the integration of AI into assistive technologies to enhance autism care.	AI is used in devices like smart glasses and robotics to support communication and social engagement for individuals with autism.	Autism, Assistive Technology, AI Integration
Pancholi et al. [28]	Review of AI techniques that assist individuals with physical disabilities, including mind-controlled exoskeletons, bionic limbs, and intelligent wheelchairs.	Exploring AI-powered assistive technologies for enhancing the independence of individuals with physical disabilities.	AI techniques like brain–computer interfaces, computer vision, and natural language processing enable advanced assistive devices.	Assistive Technology, Disability, AI Integration
Yanagawa et al. [29]	Examination of AI in diagnostic imaging for thoracic conditions, focusing on lesion detection and qualitative diagnosis.	Investigating AI’s role in thoracic diagnostic imaging, aiding radiologists in lesion detection and diagnosis.	AI enhances diagnostic accuracy, with explainable AI models being developed to assist radiologists in clinical decisions.	Medical Imaging, Diagnostics, AI Assistance
Singh and Krishnan [30]	Focus on EEG signal analysis and feature extraction, including applications in assistive technology and brain–computer interfaces.	Discussing the use of AI for EEG signal processing and its applications in neurological disease classification and assistive technology.	AI aids in processing EEG signals to enhance features for applications like brain–computer interfaces and medical diagnoses.	EEG Analysis, Brain–Computer Interface, AI Applications
de Freitas et al. [31]	A systematic review of AI of Things (AIoT) applications in assistive technology, particularly for visual impairment.	Exploring the integration of AI and IoT in assistive technology to help people with disabilities.	AI models, particularly machine learning, are used to analyze data from IoT devices, improving assistive technologies like visual aids.	AI of Things, Assistive Technology, IoT Integration
Shinohara [32]	Discussion on the potential of AI to assist in surgeries, particularly minimizing the mental and physical burden on surgeons.	Investigating AI’s role in enhancing surgical precision and reducing surgeon strain.	AI-controlled robots and computer vision can assist surgeons, improving efficiency and comfort without replacing them.	Surgery, AI Assistance, Robotics
Madahana et al. (2022) [33]	Proposed AI-based real-time speech-to-text to sign language translator for the hearing impaired in South Africa.	Developing an AI solution to aid communication between individuals with hearing impairments and the hearing population.	AI-driven speech-to-text and sign language translation systems could bridge communication gaps for the hearing impaired.	Assistive Technology, Hearing Impairment, AI Communication
Lee et al. [34]	Systematic review and meta-analysis of the effect of AI robots on the cognitive function of older adults.	Examining the role of AI-powered socially assistive robots (SAR) in enhancing cognitive function in older adults.	AI robots improve cognitive function by providing social interaction and engagement, particularly in anthropomorphic forms.	Cognitive Health, Elderly Care, AI Robots
Lee-Cheong et al. [35]	Review of new assistive technologies for dementia and mild cognitive impairment (MCI) care.	Investigating AI-driven technologies designed to support individuals with dementia and MCI and reduce caregiver burden.	AI systems help with diagnosis, monitoring, and managing dementia and MCI, offering affordable care alternatives.	Dementia Care, AI in Healthcare, Assistive Technology
Naeem et al. [36]	Analysis of AI methods like deep learning and federated learning for brain tumor diagnosis.	Focusing on AI’s role in early brain tumor diagnosis using deep and federated learning models.	AI-driven deep learning and federated learning enhance diagnostic accuracy and early detection of brain tumors.	Brain Tumor Diagnosis, AI in Medicine, Deep Learning
Alabdulkareem et al. [37]	Review of robot-assisted therapy for children with autism, focusing on AI’s role.	Exploring the use of AI-powered robots in autism therapy to improve social and cognitive skills in children.	AI robots facilitate interaction, guiding children with autism through therapeutic activities.	Autism Therapy, AI Robotics, Child Development
Dada et al. [38]	A scoping review on intelligent assistive technology for persons with dementia (PwD), especially addressing cognitive and communication impairments.	Investigating the role of AI in developing assistive technologies for cognitive and communication challenges in PwD.	AI technologies like social robots improve cognitive and communication skills, aiding PwD in daily activities.	Dementia, Cognitive Impairment, AI-Assisted Communication
Vollmer et al. [39]	Review on the use of intelligent assistive technology for people with dementia and their caregivers in the U.S.	Exploring AI’s role in reducing caregiver burden and enhancing the quality of life for people with cognitive deficits.	AI tools, including robots and sensors, provide support to caregivers and improve healthcare for older adults with dementia.	Caregiver Support, Cognitive Deficits, AI in Elderly Care

**Table 2:** AI Application Categories and Key Contributions.

AI Application Category	Study Reference	Focus	Key Results/Contribution
**AI in Robotics and Surgery**	Shinohara [32]	AI-controlled robots in surgery	AI-controlled robots assist surgeons, enhancing precision and reducing physical strain
**AI in Robotics and Surgery**	Xie et al. [26]	Robot-assisted gynecological surgery	AI improves precision and efficiency in robot-assisted laparoscopic surgeries
**AI in Robotics and Surgery**	Alabdulkareem et al. [37]	Robot-assisted therapy for children with autism	AI robots facilitate therapeutic interactions for children with autism
**AI for Medical Diagnosis**	Wei et al. [25]	Skin cancer diagnosis through AI	AI aids in image processing and molecular analysis for accurate diagnosis of skin cancer
**AI for Medical Diagnosis**	Yanagawa et al. [29]	AI in thoracic diagnostic imaging	AI enhances lesion detection in thoracic imaging, improving diagnostic accuracy
**AI for Medical Diagnosis**	Naeem et al. [36]	AI methods for brain tumor diagnosis	Deep learning improves accuracy and early detection of brain tumors
**AI for Brain–Computer Interfaces**	Eldawlatly [24]	Generative AI for BCIs	AI generates synthetic brain data to enhance BCI system performance
**AI in Assistive Tech for Disabilities**	Pancholi et al. [28]	AI-powered exoskeletons and bionic limbs	AI enables mobility and independence for individuals with physical disabilities
**AI in Assistive Tech for Disabilities**	Okolo et al. [23]	Assistive tech for the visually impaired	AI-based navigation aids support the visually impaired in mobility
**AI in Assistive Tech for Disabilities**	Iannone and Giansanti [27]	AI integration in autism care	AI supports communication and social engagement for people with autism
**AI in Cognitive Health and Aging**	Lee et al. [34]	Cognitive health and aging through AI-powered robots	AI robots enhance cognitive function and social interaction for elderly people
**AI in Cognitive Health and Aging**	Kokorelias et al. [21]	Interaction between older adults and smart technologies	Smart AI devices like virtual assistants improve elderly care through personalized interactions
**AI in Emotional and Educational Support**	Vistorte et al. [22]	Emotion recognition in educational environments	AI-driven emotion detection improves learning outcomes by assessing emotional states in students
**AI in Communication Aids**	Madahana et al. [33]	AI for speech-to-text and sign language translation	AI enhances communication for the hearing impaired through real-time speech-to-text systems
**AI for Diagnostic Imaging and Disease Monitoring**	Singh and Krishnan S [30]	EEG signal analysis for neurological diseases and assistive tech	AI improves EEG signal analysis for brain–computer interfaces and neurological diagnostics
**AI for Diagnostic Imaging and Disease Monitoring**	de Freitas et al. [31]	AI of Things (AIoT) for assistive tech in visual impairment	AI-driven IoT devices enhance assistive technology for people with visual impairments
**AI for Dementia and Cognitive Care**	Lee-Cheong et al. [35]	AI in dementia and mild cognitive impairment (MCI) care	AI assists in diagnosing, monitoring, and managing dementia and MCI
**AI for Dementia and Cognitive Care**	Dada et al. [38]	AI for people with dementia	AI-powered social robots help individuals with dementia improve communication and cognitive abilities
**AI for Emotion and Psychological Support**	Madahana et al. [33]	AI for speech-to-text and sign language translation	AI supports emotional communication for the hearing impaired by translating speech into sign language

### 3.3. Emerging Opportunities and Challenges and Key Recommendations

Table 3 presents emerging opportunities and challenges in AI applications across various domains. It highlights how AI-driven technologies can improve healthcare, assistive devices, and user experiences. Key opportunities include AI’s role in enhancing independence for older adults, improving surgical precision, and enabling better diagnostic accuracy. Additionally, AI-powered assistive technologies such as exoskeletons and bionic limbs promote accessibility and autonomy. However, significant challenges persist, such as privacy concerns, regulatory barriers, and the need for AI integration within existing medical frameworks. Other limitations include high costs, technological adaptability, and ethical considerations. These factors shape the future development and adoption of AI technologies.

**Table 3:** Emerging opportunities and challenges.

Reference	Opportunities	Challenges
Kokorelias et al. [21]	Enhances the independence and well-being of older adults, improves user experience, and optimizes technology utility over time.	Lack of customization, user-friendly interfaces, limited adoption due to communication barriers, small sample sizes, and limited diversity in study populations.
Vistorte et al. [22]	Promising applications in emotional assessment, improving pedagogical strategies, and adaptive learning environments using AI.	Accuracy, privacy concerns, cross-cultural validity, and the need for further research to refine AI models and address emerging factors.
Okolo et al. [23]	Development of multi-modal mobility assistance solutions for indoor and outdoor environments, improving daily life for visually impaired persons.	Limitations in current navigation assistive technologies, the need for more advanced solutions for navigation, and adaptation to complex environments.
Eldawlatly [24]	The potential of generative AI to improve BCI performance, augment EEG data, enhance cross-subject performance, and increase the accessibility of BCIs as assistive technology.	Limited data availability, inter-subject variability, challenges in BCI applications relying on complex brain patterns, and the need for further advancements in BCI technology.
Wei et al. [25]	AI’s role in skin cancer screening, improving diagnostic accuracy, and aiding non-dermatologists in self-screening and diagnosis.	Barriers to clinical implementation, ethical considerations, and the need for integration with existing medical practices and systems.
Xie et al. [26]	Enhancements in robotic laparoscopic surgery, improving precision and recovery times, especially in gynecological conditions.	The evolving nature of robot-assisted surgery, challenges in measuring benefits compared to traditional methods, and integration with existing practices.
Iannone and Giansanti [27]	AI’s potential to enhance communication, interaction, and social engagement for individuals with autism, with innovations in robotics and wearable devices.	Regulatory and acceptance challenges, the need for a stronger presence in healthcare, and the integration of AI with autism care are facing barriers in implementation.
Pancholi et al. [28]	AI-powered assistive technologies, like exoskeletons and bionic limbs, enable greater independence for individuals with physical disabilities, improving daily life, education, and employment opportunities.	AI systems need to be adaptable to various disabilities and scalable, but their high cost and infrastructure requirements limit accessibility.
Yanagawa et al. [29]	AI can enhance diagnostic accuracy in imaging, aiding in faster and more accurate lesion detection, and improving workflow and patient care.	The need for explainable AI in medical diagnoses is critical, and AI should be used as a tool alongside human judgment, not a replacement.
Singh and Krishnan [30]	AI-based EEG analysis enables applications in brain–computer interfaces and neurological disease classification, improving diagnosis and control for those with neurological impairments.	The complexity of EEG signal processing and the need for high computational power make it challenging to implement these technologies reliably in clinical settings.
de Freitas et al. [31]	AIoT devices can offer real-time, personalized support for individuals with disabilities, particularly for visual impairments, enhancing accessibility and quality of life.	Privacy concerns and the need for device standardization across platforms make large-scale adoption of AIoT in assistive technologies difficult.
Shinohara [32]	AI can improve surgical precision, reduce surgeon fatigue, and assist in minimally invasive procedures, enhancing patient safety.	AI cannot replace the complex, human-centered decision-making needed in surgery, and there are risks in over-relying on AI for critical decisions.
Madahana et al. (2022) [33]	AI-based real-time speech-to-sign language translation can improve communication for the hearing impaired, particularly in regions with limited access to sign language interpreters.	Limited research and adoption in African countries hinder the implementation of AI solutions for the hearing impaired, and accuracy in real-time translation remains a challenge.
Lee et al. [34]	AI robots can serve as caregivers for older adults, enhancing cognitive function and promoting social interaction, with the potential to reduce caregiver burden.	Ethical concerns, the digital literacy of older adults, and the need for more research with larger sample sizes hinder the implementation and expansion of AI-based caregiving technologies.
Lee-Cheong et al. [35]	Assistive technologies can help dementia patients and individuals with mild cognitive impairment (MCI) maintain independence, manage daily tasks, and reduce caregiver burnout.	Legal, privacy, and healthcare regulations pose barriers to the widespread adoption and integration of these technologies, which need to be addressed to ensure their effectiveness and accessibility.
Naeem et al. [36]	AI and deep learning techniques have shown promise in diagnosing brain tumors early, which can improve patient outcomes and survival rates.	Challenges include the need for high-quality data and validation of AI methods, as well as the integration of these systems into clinical workflows without overwhelming healthcare providers.
Alabdulkareem et al. [37]	Robot-assisted therapy (RAAT) can improve social interaction and therapeutic engagement for children with autism, fostering essential skills in a controlled environment.	Technology is still evolving and scaling it for widespread use in diverse settings remains a challenge, along with ensuring it meets the therapeutic needs of all children with autism.
Dada et al. [38]	Intelligent assistive devices (IATDs) offer cognitive and communication support for individuals with dementia, improving quality of life and reducing caregiver strain.	Most IATDs are still in prototype stages, and there are gaps in research and collaboration between computer engineers and healthcare practitioners to address the real-world needs of people with dementia.
Vollmer et al. [39]	Intelligent assistive technologies can reduce caregiver burden and improve healthcare services for older adults with cognitive deficits, enhancing their overall quality of life.	Socioeconomic disparities, technological literacy, and access to these technologies remain significant barriers to their widespread adoption among vulnerable populations.

From the analysis of the studies and the challenges highlighted, both direct and indirect recommendations for future directions emerge, as outlined in Table 4 and Table 5.

**Table 4:** Emerging direct recommendations.

Study	Recommendation	Focus	Key Action
[21]	Enhancing Personalization and Customization	Explores the coadaptation process between older adults and smart technologies, recommending increased customization for better integration.	Develop adaptive systems that personalize AI responses based on user preferences, health conditions, and environment.
[22]	Improving User Interfaces	Investigates AI in emotional recognition for educational settings, focusing on improving interfaces for better user engagement.	Simplify interfaces to ensure elderly and disabled users can interact easily with devices.
[25]	Improving Diagnostic Accuracy and Efficiency	Highlights AI’s role in improving skin cancer diagnosis through enhanced image analysis, recommending more advanced diagnostic tools.	Enhance AI models to improve diagnostic precision in fields like oncology and neurology (e.g., skin cancer detection, EEG analysis).
[29]	Improving Diagnostic Accuracy and Efficiency	Discusses the use of AI in thoracic diagnostic imaging, recommending more precise diagnostic models.	Develop more accurate AI diagnostic models for imaging in fields like thoracic diagnostics.
[28]	Increasing Accessibility of Devices	Focuses on AI-powered exoskeletons, recommending strategies to ensure these devices are accessible and affordable, especially for underserved populations.	Implement strategies to make AI devices affordable and accessible, particularly in underserved regions.
[33]	AI-Driven Mobility Assistance for the Visually Impaired	Develops speech-to-text AI for the hearing impaired, offering a potential framework for assistive navigation tools for the visually impaired.	Create AI-driven multi-modal systems for visually impaired individuals to navigate both indoor and outdoor environments effectively.

**Table 5:** Emerging indirect recommendations.

Study	Recommendation	Focus	Key Actions
[24]	Developing Ethical and Regulatory Frameworks	Addressing privacy, data security, and ethical concerns.	Create comprehensive regulatory standards ensuring data privacy, security, and ethical use of AI in healthcare and education.
[28]	Promoting Interdisciplinary Collaboration	Combining expertise from multiple fields.	Foster collaboration between AI researchers, healthcare professionals, and policymakers to ensure the development of holistic solutions.
[38]	Increasing Public Awareness and Trust	Promoting the adoption of AI-assisted technologies.	Launch educational campaigns and transparency initiatives to build public trust and awareness regarding the safety and benefits of AI technologies.
[21]	Facilitating Cross-Cultural Adaptation	Ensuring technologies are culturally appropriate.	Adapt AI solutions to diverse cultural contexts and healthcare systems to increase their global applicability.
[22]	Standardizing AI Devices	Ensuring interoperability between different platforms.	Establish industry standards for AI devices to ensure compatibility and integration with various systems and technologies.

## 4. Discussion

The discussion is divided into four sections, each providing a structured analysis of the study’s findings. Section 4.1 summarizes the key highlights and main outcomes of the review. Section 4.2 discusses trends, opportunities, and challenges in AI-driven assistive technologies, offering actionable recommendations. Section 4.3 builds on these recommendations, discussing future perspectives and recent studies that advance the field. Section 4.4 outlines the study’s limitations.

### 4.1. Summary and Added Value

This NRR examined AI’s role in assistive technologies (ATs), analyzing 19 studies with a structured methodology [20]. Given AI’s rapid evolution, the first added value is that the NRR captures key trends and emerging themes, and categorizes findings, while also highlighting gaps in the field, which helps guide future research and development directions. A second added value is the identification of key themes, including AI-powered devices supporting elderly autonomy, such as smart wheelchairs and exoskeletons [21,28], and AI-driven tools enhancing social interaction for individuals with autism [27]. These examples show AI’s practical applications in improving autonomy and social engagement, aligning with ongoing recommendations for personalized, non-invasive interventions. Additionally, AI-assisted imaging and robotics are advancing skin cancer detection and EEG-based brain–computer interfaces [24,25,29], demonstrating AI’s potential in enhancing diagnostics and cognitive support. The third added value is the classification of research into broad clusters, enabling a comparative analysis of AI applications in AT. This approach provides a clearer understanding of how AI enhances independence and well-being [21,27], while also highlighting challenges such as personalization, accessibility, and privacy concerns [25,29]. This analysis is particularly relevant given the rapid expansion of the AT market, projected to grow from USD 23 billion in 2023 to USD 37 billion by 2033, driven by AI innovations in mobility, sensory, and cognitive support [40,41,42]. Governments are fostering accessibility, with North America leading, followed by Europe and Asia-Pacific [40,41,42].

### 4.2. Discussion on AI in Assistive Technologies: Evolving Trends, Themes, Opportunities Barriers, and Key Recommendations

The overview aligned with the objectives, highlighting trends in scientific production, emerging themes, and key opportunities and challenges. Regarding the trend, bibliometric analysis shows a sharp rise in AI-integrated AT research, especially in the last five years, shifting from theoretical studies to real-world applications. AI is increasingly advancing mobility aids, cognitive support, and accessibility tools, surpassing traditional AT research. A growing number of reviews indicate a maturing field focused on synthesizing knowledge and refining AI-driven solutions. Concerning AI Themes, AI’s impact spans various domains. In Assistive Technologies and Disability Support, AI-driven exoskeletons and smart navigation systems enhance mobility [23,28], while robots aid autism therapy [37]. Healthcare and Medical Diagnostics benefit from AI-enhanced imaging for skin cancer and brain tumor detection [25,36]. Cognitive Health and Aging sees AI-powered robots improving elderly care and social engagement [21,34]. AI also supports Emotional Support and Education, aiding learning and communication for individuals with hearing impairments [22,33]. Robot-Assisted Surgery and Healthcare Robotics enhance surgical precision and autism therapy [26,37], while brain–computer interfaces (BCIs) enable individuals with severe disabilities to communicate [24]. Finally, Dementia and Psychological Support leverages AI-driven social robots to improve patient interaction and ease caregiver burden [38]. Opportunities and challenges emerged: AI offers transformative potential in AT but faces key barriers. In elderly care, AI-powered robots promote independence, though customization remains a challenge [21]. Education benefits from adaptive AI-driven learning but faces privacy concerns [22]. AI aids assistive technologies for the visually impaired, yet struggles in complex environments [23]. BCIs show promise but suffer from variability in brain patterns [24]. AI-driven Medical Diagnostics improve accuracy but raise ethical and clinical concerns [25,36]. Robot-assisted surgery enhances precision but must demonstrate superiority over traditional methods [26]. AI supports autism therapy, though scalability remains an issue [37]. Dementia care benefits from AI solutions but requires collaboration between developers and healthcare professionals for real-world implementation [38].

The analysis stimulated key recommendations for AI in AT. AI holds significant promise for improving healthcare and quality of life, but overcoming technical, ethical, and accessibility barriers is essential. Direct recommendations (Table 4) focus on refining AI functionality, personalization, user interfaces, and diagnostics for developers, researchers, and healthcare providers. Indirect recommendations (Table 5) emphasize ethical guidelines, regulatory frameworks, and cultural adaptation, alongside public awareness, trust, and standardization. Together, these recommendations provide a roadmap for AI development and adoption, ensuring its transformative potential is fully realized.

### 4.3. Discussion and Future Perspectives on AI in Assistive Technologies

The direct and indirect recommendations (Table 4 and Table 5) have been used as a framework to stress the strongly needed actions based on the reviewed studies and to focus on recent primary studies to investigate how they align with them and, therefore, the future perspectives.

#### 4.3.1. Overcoming Barriers in AI-Powered Assistive Technologies

AI-driven ATs have the potential to improve the lives of individuals with disabilities, the elderly, and those with complex healthcare needs. However, several challenges still limit their widespread adoption, particularly cost, scalability, and ethical concerns. The financial burden of developing and maintaining AI technologies remains high, as highlighted by Kokorelias et al. [21], making accessibility difficult, especially for advanced solutions like AI-powered exoskeletons. Additionally, many low-resource settings lack the necessary infrastructure and expertise to integrate AI effectively.

Beyond financial constraints, socioeconomic barriers further limit AI’s reach. As noted by Pancholi et al. [28], high costs and limited digital access make AI-powered solutions inaccessible in underserved regions, highlighting the need for affordable and scalable alternatives. Another critical issue is privacy and ethical considerations, particularly in healthcare applications. Protecting sensitive data requires compliance with strict regulations like GDPR and HIPAA, as emphasized by Eldawlatly et al. [24]. Ensuring transparency through explainable AI (XAI) can help build trust, while cross-cultural adaptation remains key to making AI solutions effective across diverse populations [21].

Equally important is addressing algorithmic bias and data inequities. AI systems trained on non-representative datasets can reinforce disparities in diagnosis and treatment, potentially worsening healthcare inequalities. As highlighted by Pancholi et al. [28], ensuring diverse, inclusive data and fostering global data-sharing initiatives are essential steps toward developing fair and effective AI-powered assistive technologies.

Despite these challenges, AI continues to show immense promise. By prioritizing affordability, accessibility, and ethical safeguards, AI-driven solutions can become more equitable and impactful, ultimately enhancing the quality of life for a wider range of individuals.

#### 4.3.2. Emerging Directions in AI-Powered Assistive Technologies: Insights from Recent Studies

In analyzing future directions, it is also strategic to examine how cutting-edge recent studies are moving in relation to the recommendations. Sixteen studies have been selected [43,44,45,46,47,48,49,50,51,52,53,54,55,56,57,58,59,60,61,62,63,64,65,66,67,68] for their contributions in this direction, with the aim of observing and analyzing their impact on this field.

Fernandes et al. [43] explore computational approaches to generate inclusive image paragraphs for the visually impaired, enhancing accessibility through improved image captioning technologies. This directly supports the recommendation to refine AI functionality in assistive tech, with a particular focus on inclusivity and user-centric design. Wang et al. [44] investigate wearable EEG neurofeedback for children with autism using machine learning algorithms, which aligns with the recommendation for personalized and non-invasive therapeutic interventions. This study highlights the potential for AI to support earlier interventions in autism therapy, contributing to the development of tailored solutions. Similarly, Ferreira de Oliveira Neto et al. [45] focus on software requirements for image captioning for visual impairments, providing insights into creating more effective and context-sensitive software, which is in line with recommendations for accessibility improvements and customized design in assistive technologies. Değerli and Özata Değerli [46] presents a case report on using ChatGPT 4 in occupational therapy for mild cognitive impairment, reinforcing the value of AI in cognitive rehabilitation and therapeutic interventions. This aligns with recommendations to integrate AI into cognitive health solutions. Rafi et al. [47] offer an open dataset for Bangladeshi paper currency detection, contributing to AI-powered financial assistance for the visually impaired, supporting advancements in assistive technologies and accessibility for marginalized groups. In terms of educational applications, Jackson et al. [48] investigate medical students’ perceptions of AI in medical education, supporting the recommendation to incorporate AI training into healthcare curricula. This ensures that future healthcare professionals are equipped to use AI tools in practice. Similarly, Rupp et al. [49] highlight orthopedic surgeons’ positive outlook on AI integration, showing readiness for AI adoption in clinical practice, which is vital for the effective implementation of AI solutions. Bonteanu et al. [50] implement a high-accuracy neural network for real-time pupil detection, strengthening assistive technologies for individuals with visual impairments. This improves real-time interaction with AI systems, aligning with recommendations to refine user interfaces for more effective and personalized assistive solutions. Lagos et al. [51] provide evidence on the impact of robotic assistive technology for cerebral palsy, supporting recommendations for robotics in healthcare. The study demonstrates the potential for robotics to enhance physical rehabilitation, which aligns with the call for increased integration of robotics in therapeutic settings. Meanwhile, Redahan and Kelly [52] and Fins and Shulman [53] explore the ethical implications of AI in healthcare, specifically in relation to mental capacity legislation and neuroethics. These studies contribute to the need for ethical guidelines and responsible AI use in clinical settings. Reynolds and Tejasvi [54] examine ChatGPT’s potential for responding to patient queries and creating patient resources, which aligns with recommendations for AI-driven patient education and communication. This highlights the role of AI in improving healthcare accessibility and patient engagement. Lee et al. [55] validate a deep learning model for chest X-ray interpretation, advancing AI’s role in diagnostic imaging and supporting recommendations for its integration into healthcare diagnostics. The work of Dlugatch et al. [56] investigates obstetricians’ perspectives on AI-driven clinical decision-making, emphasizing the necessity of AI in clinical decision support systems, particularly in maternal health. Hesselmans et al. [57] examine stress-based interventions for people with intellectual disabilities and their caregivers, reinforcing the integration of AI-driven interventions to improve quality of life. Ventura et al. [58] co-design an interactive AI system for post-stroke rehabilitation, supporting the need for human-centered AI design in rehabilitation. This aligns with the recommendation to prioritize user-centered design in AI development. Smith et al. [59] review the risks and rewards of AI in assistive technology, providing a comprehensive analysis of the challenges and opportunities, which directly aids in refining recommendations for AI’s role in assistive tech. Guo [60] explores the impact of assistive technology on psychological health in Japan, offering a cross-cultural perspective on AI in assistive tech and broadening the scope of its application. Schicktanz et al. [61] address AI-assisted ethics in designing assistive technologies, reinforcing the need for responsible AI development frameworks. Hallowell et al. [62] discuss ethical issues raised by AI in rare disease diagnosis, contributing to the discussion on ethical AI use in underserved areas of healthcare. Bhola and Vishwakarma [63] review vision-based human activity recognition in indoor environments, expanding AI’s role in enhancing accessibility and mobility for individuals with visual impairments. Javed et al. [64] investigate explainable AI for cognitive health assessment, contributing to the growing need for transparent and trustworthy AI applications, especially in cognitive health. Kang et al. [65] study nurses’ perceptions of care robots during COVID-19, strengthening the case for robotic and AI-based care technologies in healthcare settings, and supporting adoption in care environments. Muthu [66] highlights the utility of AI in improving research processes, particularly in systematic reviews, aligning with recommendations for AI’s role in clinical research. Civaner et al. [67] focus on AI in medical education, emphasizing the need for AI training in medical curricula to ensure healthcare professionals are prepared for AI tools. Lastly, Tiwary and Mahapatra [68] develop an image captioning method for the blind, directly contributing to improved accessibility and inclusivity in assistive tech.

### 4.4. Limitations

The study, designed as a narrative review, presents certain limitations related to its methodology and the inclusion/exclusion criteria adopted. The decision to exclude conference proceedings may result in missing emerging research or recent developments that are still in preliminary phases and not yet formally published. Furthermore, by excluding local studies or guidelines written in languages other than English, the review is confined to internationally published literature. This may lead to the omission of region-specific insights or contextually relevant practices, potentially limiting the understanding of variations in clinical approaches and treatment protocols across different cultural or healthcare settings.

## 5. Conclusions

The integration of AI into assistive technologies is rapidly transforming sectors such as healthcare, mobility, autism support, and medical diagnosis. AI is enhancing elderly independence through smart devices that improve communication, mobility, and cognitive abilities. At the same time, traditional assistive technologies like smart wheelchairs and exoskeletons are being optimized with AI for more personalized performance. AI also plays a crucial role in fostering social interaction and cognitive skills for children with autism. In healthcare, AI improves diagnostic accuracy in imaging and supports robotic surgeries, all contributing to better outcomes.

While the potential of AI-powered assistive technologies is vast, offering improved autonomy and quality of life for the elderly and individuals with disabilities, several challenges remain. Issues such as technology customization, digital literacy barriers for the elderly, and privacy concerns must be addressed for these technologies to be widely adopted. Importantly, AI itself is now emerging as an assistive technology, expanding beyond traditional tools like mobility devices and hearing aids. AI-driven systems are capable of not only supporting basic tasks but also learning from user behavior, adapting to changing needs, and offering highly personalized solutions. This shift marks a significant evolution in the nature of assistive technologies, transitioning from static tools to dynamic, adaptive systems.

However, this progress is not without its challenges. The growing complexity of AI systems raises strategic concerns related to scalability, cost, and accessibility, particularly for low-resource settings. Additionally, ethical issues such as data privacy, algorithmic fairness, and ensuring the reliability of AI in sensitive areas like healthcare and caregiving must be carefully navigated. Addressing these challenges is critical to fully unlocking the potential of AI in transforming assistive technologies and ensuring they benefit all users equitably.

## Data Availability

No new data were created. Data sharing is not applicable.

## Supplementary Materials

The following supporting information can be downloaded at: https://www.mdpi.com/article/10.3390/healthcare13050556/s1, Text S1. Analitical summary; Text S2. Ranking results based on the methodology of quality assessment in the methods; Table S1. Output from the qualification process; Table S2. ANDJ checklist.
