# Supplementary material for: Integrating AI and Assistive Technologies in Healthcare: Insights from a Narrative Review of Reviews
Source: Healthcare (Basel). 2025 Mar 4;13(5):556. doi: 10.3390/healthcare13050556 (PMC11898476; doi:10.3390/healthcare13050556)
Supplement: Supplementary file 1 [file healthcare-13-00556-s001.zip › healthcare-3456896-supplementary.pdf]

Text S1. Analitical summary

[21] Longitudinal Coadaptation of Older Adults With Wearables and Voice-Activated Virtual Assistants: Scoping Review

This scoping review explores how older adults adapt to and co-evolve with wearable technologies and voice-activated virtual assistants. It assesses how such technologies have been integrated into daily routines, improving health management, monitoring, and autonomy. The review underscores the potential of wearables and AI assistants in promoting independent living, mental well-being, and engagement with healthcare providers, while also highlighting the need for ongoing support and personalized adaptation to individual user preferences.

[22] Integrating Artificial Intelligence to Assess Emotions in Learning Environments: A Systematic Literature Review

This systematic review investigates how AI is used to assess and interpret emotional states in educational settings. The study highlights the potential of AI systems to analyze student responses, adapt educational content to emotional states, and personalize learning experiences. This could significantly benefit learners with cognitive disabilities or emotional challenges, improving educational outcomes by providing a more responsive and individualized approach.

[23] Assistive Systems for Visually Impaired Persons: Challenges and Opportunities for Navigation Assistance

Focusing on assistive navigation systems for the visually impaired, this paper examines how AI and sensor technologies support mobility. The challenges outlined include environmental unpredictability and the need for seamless integration with existing infrastructure. However, the paper also emphasizes the potential for AI-driven navigation aids, such as smart glasses and wearable devices, to significantly enhance independence and mobility for visually impaired individuals.

[24] On the Role of Generative Artificial Intelligence in the Development of Brain-Computer Interfaces

This paper discusses the transformative role of generative AI in the development of brain-computer interfaces (BCIs). Generative AI techniques, particularly deep learning, are enhancing the accuracy and responsiveness of BCIs, enabling users with motor impairments to control prosthetics or communicate. The study highlights how generative AI improves signal decoding and personalization of BCI applications, offering new possibilities for users to interact with their environment in more intuitive and adaptive ways.

[25] Artificial Intelligence and Skin Cancer

This paper explores the application of AI in diagnosing and monitoring skin cancer. Through image analysis, AI systems can identify melanoma and other skin conditions with high accuracy, potentially surpassing traditional diagnostic methods. The integration of AI in dermatology offers a promising avenue for early detection, enabling more effective treatment plans for patients with skin cancer.

[26] Robot-Assisted Laparoscopic Surgery in Gynecology: An Evolving Assistive Technology

This study discusses the integration of AI in robot-assisted laparoscopic surgery within the field of gynecology. AI-powered robots enhance the precision of surgical procedures, reducing recovery time and improving patient outcomes. The paper focuses on the ongoing evolution of this technology, highlighting both the current benefits and future potential for expanding AI's role in minimally invasive surgeries.

[27] Breaking Barriers-The Intersection of AI and Assistive Technology in Autism Care: A Narrative Review

This narrative review investigates how AI can improve autism care through personalized interventions, including behavioral therapy and communication aids. The authors examine current AI-based tools that help children with autism improve social skills, reduce communication barriers, and promote independent functioning. The paper advocates for the growing role of AI in creating more effective, individualized care strategies for individuals with autism.

[28] Use of Artificial Intelligence Techniques to Assist Individuals with Physical Disabilities

The review explores AI techniques designed to assist people with physical disabilities, ranging from mobility aids to assistive robots. AI is being used to enhance the functionality of devices such as smart prosthetics and exoskeletons, providing users with greater independence and the ability to perform everyday tasks with less reliance on caregivers.

[29] New Trend in Artificial Intelligence-Based Assistive Technology for Thoracic Imaging

This paper highlights advancements in AI-based assistive technology within thoracic imaging. AI tools improve the accuracy and speed of diagnosing chest conditions such as lung cancer and tuberculosis. The research demonstrates how AI-powered imaging systems can enhance diagnostic workflows, enabling early detection and more targeted treatment options.

[30] Trends in EEG Signal Feature Extraction Applications

This article examines the latest trends in EEG signal processing, focusing on AI-based techniques for extracting relevant features from EEG signals. These methods are used in the diagnosis of neurological conditions such as epilepsy, sleep disorders, and cognitive impairments. AI's role in this field is growing, with significant potential for improving diagnostic accuracy and creating personalized treatment regimens.

[31] Artificial Intelligence of Things Applied to Assistive Technology: A Systematic Literature Review

This systematic review discusses the integration of AI and the Internet of Things (IoT) in assistive technology. AI-driven IoT devices are transforming the landscape by offering real-time monitoring and personalized interventions, which are crucial for individuals with disabilities. The paper provides an overview of various IoT-enabled devices that can improve daily living and enhance safety for users with different needs.

[32] Surgery Utilizing Artificial Intelligence Technology: Why We Should Not Rule It Out

This paper examines the potential benefits of incorporating AI into surgery, particularly in improving precision, minimizing human error, and supporting complex decision-making. AI tools are already assisting in preoperative planning, intraoperative guidance, and postoperative care, showing promise in enhancing surgical outcomes and reducing recovery times.

[33] A Proposed Artificial Intelligence-Based Real-Time Speech-to-Text to Sign Language Translator for South African Official Languages for the COVID-19 Era and Beyond: In Pursuit of Solutions for the Hearing Impaired

This study proposes a real-time AI-based system that translates spoken language into text and sign language, facilitating communication for the hearing-impaired community. The system is particularly focused on South African official languages, addressing the diverse needs of users and improving accessibility in communication across various domains, such as education and healthcare.

[34] The Effect of Cognitive Function Health Care Using Artificial Intelligence Robots for Older Adults: Systematic Review and Meta-analysis

This systematic review assesses the effectiveness of AI-powered robots in promoting cognitive health among older adults. The study finds that such robots can assist in cognitive training, memory support, and overall mental well-being, showing promise in reducing the impact of cognitive decline and dementia.

[35] New Assistive Technologies in Dementia and Mild Cognitive Impairment Care

This review focuses on recent advances in assistive technologies for dementia and mild cognitive impairment. It discusses AI-driven tools, such as memory aids and interactive robots, which help manage daily activities, track medication adherence, and provide companionship, thus improving the quality of life for individuals with cognitive impairments.

[36] A Comprehensive Analysis of Recent Deep and Federated Learning-Based Methodologies for Brain Tumor Diagnosis

This analysis explores the role of deep learning and federated learning in the diagnosis of brain tumors. AI techniques applied to medical imaging help improve the accuracy of early tumor detection, while federated learning offers a privacy-preserving method for training AI models across decentralized healthcare data.

[37] A Systematic Review of Research on Robot-Assisted Therapy for Children with Autism

The review investigates robot-assisted therapy for children with autism, highlighting how robotic systems can aid in behavioral therapy and communication. These systems provide interactive, engaging, and structured environments for children, promoting skill development and improving social interactions.

[38] Intelligent Assistive Technology Devices for Persons with Dementia: A Scoping Review

This scoping review covers various intelligent assistive devices designed for individuals with dementia, such as memory aids, GPS trackers, and communication tools. The study emphasizes the positive impact of these technologies in supporting daily living and assisting caregivers in managing dementia-related challenges.

[39] Emerging Issues of Intelligent Assistive Technology Use Among People with Dementia and Their Caregivers: A U.S. Perspective

This paper explores the emerging issues surrounding the use of intelligent assistive technologies in dementia care, focusing on the U.S. context. It highlights the benefits of AI tools in improving patient independence, safety, and well-being, while also addressing challenges such as cost, accessibility, and caregiver training.

Text S2. Ranking results based on the methodology of quality assessment in the methods

In Table S1, the studies are presented with the output of the qualification process. The studies are anonymized and do not correspond to the reference indicated in the review."

Explanation:

In this section (S.2), the results are ranked based on the methodology used to assess the quality of the studies. This means the studies are organized to reflect their reliability or rigor according to the criteria set out in the methodology section.

Table S.1 provides an overview of the studies considered, showing the results of the qualification process applied to each of them. However, the studies are anonymized, meaning their identities are not disclosed, and they are not linked to the reference listed in the review to ensure a more objective and neutral analysis.

In other words, the purpose is to present the data without external influences related to the studies' origins, focusing solely on the quality of their content.

Table S1. Output from the qualification process

Study	N 1	N 2	N 3	N 4	N5
**Study 1**	4	4	3	5	5
**Study 2**	5	5	5	3	5
**Study 3**	4	4	3	5	3
**Study 4**	5	5	5	5	4
**Study 5**	4	5	5	4	5
**Study 6**	4	4	5	5	4
**Study 7**	4	5	5	3	3
**Study 8**	5	4	5	3	3
**Study 9**	3	5	3	3	5
**Study 10**	4	5	3	5	5
**Study 11**	5	5	4	4	3
**Study 12**	5	3	5	5	3
**Study 13**	3	3	3	4	5
**Study 14**	5	5	4	5	4
**Study 15**	5	5	5	4	4
**Study 16**	3	5	4	5	4
**Study 17**	4	5	5	5	5
**Study 18**	5	5	4	5	5
**Study 19**	5	5	3	5	5

Table S2. ANDJ checklist.

Section/topic	#	Checklist item	Reported on page
**TITLE**			
title	1	Identify the report as a Narrative Review of …	**Pag. 1**
**ABSTRACT**			
	2	Provide an unstructured summary including, as applicable: background, objective, brief summary of narrative review and implications for future research, and clinical practice or policy development.	**Pag. 1-2**
**INTRODUCTION**			
Rationale/background	3	Describe the rationale for the review in the context of what is already known.	**Pag. 1-2**
Objectives	4	Specify the key question(s) identified for the review topic.	**Pag. 2**
**METHODS**			
Research selection	5	Specify the process for identifying the literature search (eg, years considered, language, publication status, study design, and databases of coverage).	**Pag. 6**
**DISCUSSION/SUMMARY**	**N**	**Checklits item**	**ACTION**
**Narrative**	**6**	Discuss: 1) research reviewed including fundamental or key findings, 2) limitations of research reviewed, and 3) need for future research.	**Pag. 6-17**
Summary	**7**	Provide an overall interpretation of the narrative review in the context of clinical practice for health professionals, policy development and implementation, or future research.	**Pag. 17-22**
